# Association between lifelines diet score with odds of nonalcoholic fatty liver disease and some novel anthropometric indices among adults: a case–control study

**DOI:** 10.3389/fnut.2024.1523651

**Published:** 2024-12-11

**Authors:** Sahar Asiaei, Mohammad Sharif Sharifani, Bijan Ghobadian, Ghazal Baghdadi, Fereshteh Biglari, Mehran Rahimlou

**Affiliations:** ^1^Department of Exercise Physiology, Central Tehran Branch, Islamic Azad University, Tehran, Iran; ^2^Amir al-Momenin and Sahib al-Zaman Hospital, Isfahan University of Medical Science, Isfahan, Iran; ^3^Department of Internal Medicine, School of Medicine, Vali-e-Asr Hospital, Zanjan University of Medical Sciences, Zanjan, Iran; ^4^Department of Nutrition, School of Public Health, Iran University of Medical Sciences, Tehran, Iran; ^5^Educational Department, Zanjan University of Medical Sciences, Zanjan, Iran; ^6^Department of Nutrition, School of Public Health, Zanjan University of Medical Sciences, Zanjan, Iran

**Keywords:** nonalcoholic fatty liver disease, lifelines diet score, dietary quality, visceral adiposity index, case–control study

## Abstract

**Background:**

Nonalcoholic Fatty Liver Disease (NAFLD) is a prevalent condition strongly associated with poor dietary habits and obesity. The Lifelines Diet Score (LLDS), a measure of adherence to a health-promoting diet, may reduce the risk of NAFLD. This study investigates the association between LLDS and NAFLD risk, as well as its relationship with novel anthropometric indices in adults.

**Methods:**

This case–control study included 180 NAFLD patients and 250 controls aged 20–65 years from Valiasr Hospital, Zanjan, Iran. Dietary intake was assessed using a validated 147-item food frequency questionnaire, and LLDS was calculated by scoring food groups according to dietary guidelines. Anthropometric measurements included Body Mass Index (BMI), Waist Circumference (WC), A Body Shape Index (ABSI), Body Roundness Index (BRI), and Visceral Adiposity Index (VAI). Logistic regression models estimated the odds ratios (ORs) for NAFLD across LLDS quartiles.

**Results:**

Participants in the highest LLDS quartile had significantly reduced odds of NAFLD compared to those in the lowest quartile (OR = 0.49; 95% CI: 0.30–0.65; *p* < 0.001). Gender-specific analysis revealed that LLDS had a stronger inverse association with NAFLD in females (OR = 0.45; 95% CI: 0.29–0.64) than in males (OR = 0.63; 95% CI: 0.40–0.79). LLDS was inversely associated with VAI (*β* = −1.14; 95% CI: −2.89, −0.3; *p* = 0.036), but no significant associations were observed with ABSI or BRI.

**Conclusion:**

Higher LLDS scores are associated with a lower risk of NAFLD and reduced visceral adiposity, particularly in females. These findings highlight the importance of improving dietary quality as a preventive strategy for NAFLD.

## Introduction

NAFLD is a significant global health concern, affecting approximately 25% of the population worldwide (1). In Iran, the prevalence of NAFLD is estimated to range from 10 to 30%, varying by population and diagnostic methods (2). NAFLD is characterized by excessive fat accumulation in the liver among individuals who consume little or no alcohol. Its spectrum ranges from simple hepatic steatosis to nonalcoholic steatohepatitis (NASH), which can progress to fibrosis, cirrhosis, and hepatocellular carcinoma (3). The rising prevalence of NAFLD parallels the global increase in obesity, metabolic syndrome, and type 2 diabetes, making it a leading cause of liver-related morbidity and mortality (4). Identifying modifiable lifestyle factors, particularly dietary habits, is essential for NAFLD prevention and management given its complex pathophysiology and limited therapeutic options (5).

Dietary quality has emerged as a key modifiable factor influencing NAFLD progression (6). Diets high in refined sugars, saturated fats, and processed foods are associated with increased liver fat accumulation, oxidative stress, and inflammation all of which contribute to the pathogenesis of NAFLD (7). Conversely, diets rich in fruits, vegetables, whole grains, and healthy fats, such as the Mediterranean diet, have shown promise in reducing liver fat and improving liver function markers (8).

The Lifelines Diet Score (LLDS) is a dietary quality index developed to assess adherence to a balanced diet based on health-promoting and health-compromising food groups (9). Health-promoting groups include fruits, vegetables, whole grains, legumes, and unsweetened dairy, while health-compromising groups include red and processed meats, sugar-sweetened beverages, and butter (10). This score is based on the 2015 Dutch dietary guidelines and is designed to evaluate overall dietary quality in a manner that is both scientifically validated and easily applicable. Furthermore, this framework assesses adherence to dietary recommendations in a way that is not only scientifically robust but also culturally adaptable, making it suitable for diverse populations (11).

Furthermore, the LLDS has been validated in large cohort studies, showing strong associations with cardiometabolic health outcomes, including lower risks of obesity, hypertension, and type 2 diabetes (11, 12). These findings highlight its potential value in exploring complex, multifactorial conditions such as NAFLD. Despite its established relevance in other health contexts, the application of LLDS in liver health, particularly in relation to NAFLD, remains insufficiently explored. This research gap emphasizes the need to investigate the role of LLDS in improving dietary quality and its potential protective effects against NAFLD, especially in populations with distinct dietary habits and risk factors.

In addition to dietary quality, various anthropometric indices BMI, waist circumference, and novel measures like the VAI and BRI are linked to NAFLD risk. These indices capture distinct aspects of body composition, particularly visceral fat, which plays a key role in liver fat accumulation and the metabolic disturbances associated with NAFLD (13). Exploring the relationship between LLDS and these novel anthropometric indices may offer valuable insights into how dietary patterns influence body composition and, consequently, NAFLD risk.

This case–control study investigates the association between LLDS and NAFLD risk, adjusting for established risk factors. Furthermore, it explores the relationship between LLDS and novel anthropometric indices to better understand how dietary quality may influence NAFLD.

## Method

### Study design and population

The study was conducted with the approval of the Research Council and the Ethics Committee of Zanjan University of Medical Sciences, Zanjan, Iran (Ethical code: IR.ZUMS.REC.1403.189). This hospital-based case–control study was carried out at Valiasr Hospital, Zanjan, Iran, between 2023 and 2024. Participants were recruited from newly diagnosed NAFLD patients and controls who attended the Gastroenterology and Liver Clinic at Valiasr Hospital. Of 516 eligible individuals, 450 agreed to participate, resulting in a response rate of 87.2%.

To ensure data quality and minimize confounding, we excluded participants with long-term dietary modifications, chronic illnesses unrelated to NAFLD (e.g., kidney disorders, cardiovascular disease, thyroid dysfunction, or autoimmune diseases), or implausible energy intakes (<700 kcal/day or > 4,500 kcal/day). After applying these criteria, a total of 430 participants (180 cases and 250 controls), aged 20–65 years, were included in the final analysis.

The case group consisted of newly diagnosed NAFLD patients randomly selected from referrals to the Gastroenterology and Liver Clinic at Valiasr Hospital. NAFLD diagnosis was confirmed by a gastroenterologist based on a controlled attenuation parameter (CAP) score exceeding 270 dB/m. Diagnostic criteria included chronically elevated liver enzymes, absence of alcohol use, and the presence of a fatty liver detected by ultrasound (grades II and III) (14), with exclusion of alternative causes of liver disease. Additionally, patients underwent Fibroscan examinations, and those with a CAP score above 237 dB/m and a fibrosis score above 7 were diagnosed with non-alcoholic steatohepatitis.

Control participants were selected based on normal liver function tests and ultrasound results, indicating no signs of fatty liver disease. Exclusion criteria for both groups included a history of kidney or liver disorders (e.g., Wilson’s disease, autoimmune liver disease, hemochromatosis, viral hepatitis, or alcoholic liver disease), cardiovascular disease, malignancy, thyroid dysfunction, autoimmune disease, or unusual caloric intake reports (<700 kcal/day or > 4,500 kcal/day). Written informed consent was obtained from all participants before enrollment in the study.

### Anthropometric assessment

Weight was measured to the nearest 0.1 kg using a digital scale (Seca 808, Germany), and height was measured to the nearest 0.1 cm using a wall-mounted stadiometer (Seca, Germany). BMI was calculated using the standard formula: weight (kg) divided by height (m^2^). Waist circumference was measured at the narrowest point between the lower rib margin and the iliac crest. We used from a standard formula for ABSI calculation (15):

ABSI=WC/[(BMI)∧ (2/3) × (height)∧ (1/2)]

Also, BRI (16) and VAI (17) were calculated with following formulas:

BRI = BRI = 364.2–365.5 × √(1 - (waist circumference (m) / (2 × height (m)))^2)

Men: VAI = [WC/39.68 + (1.88 × body mass index [BMI])] × [triglycerides (TG) /1.03] × (1.31/HDL); women: VAI = [WC/36.58 + (1.89 × BMI)] × (TG/0.81) × (1.52/HDL)

### Assessment of dietary intake

Dietary intake was assessed using a semi-quantitative Food Frequency Questionnaire (FFQ) consisting of 147 food items consumed over the previous year. Participants reported their consumption frequency for each item as daily, weekly, or monthly, which was then converted to daily gram intakes. Nutrient intake was calculated using N4 software (version 4.0)^1^, customized with a database of Iranian foods. The FFQ’s high validity and reliability ensured the accuracy of dietary assessments (18).

### Lifelines diet score

The LLDS is a dietary quality index developed by Vinke et al. to rank individuals based on their relative food quality (11). The LLDS, aligned with the 2015 Dutch dietary guidelines, assesses dietary quality by incorporating nine health-promoting food groups (vegetables, fruits, whole grains, legumes and nuts, fish, oils and soft margarine, unsweetened dairy, coffee, and tea) and three health-compromising food groups (red and processed meats, butter and hard margarine, and sugar-sweetened beverages).

To calculate the LLDS score, we adjusted for energy intake by expressing food consumption in grams per 1,000 kcal. Participants were then categorized into five quintiles based on their food intake. The highest intake within the positive food groups was assigned a score of 5, while the lowest intake was assigned a score of 1. Also, the highest intake within the negative food groups was assigned the lowest score of 1, while the lowest intake was assigned the highest score of 5. The study’s results were summarized using the LLDS score, which ranges from 12 to 60 and reflects the combined scores of all 12 food groups (11, 19).

To examine the relationship between the LLDS and the risk of NAFLD, participants were divided into quartiles based on their LLDS scores. Quartiles were chosen to allow for meaningful comparisons across different levels of dietary quality while ensuring a sufficient number of participants in each group for statistical analysis. The quartile cutoffs were determined based on the distribution of LLDS scores within the study population, following methods commonly used in similar dietary studies to facilitate comparability and interpretability (12).

### Assessment of nondietary exposures

A trained interviewer administered all questionnaires to ensure accurate responses from participants. Demographic data were collected through a comprehensive questionnaire, while physical activity levels were assessed using the validated International Physical Activity Questionnaire (IPAQ). Laboratory test results from the past month were extracted from participants’ medical records.

### Statistical analysis

Data analysis was performed using SPSS software (version 20). Normality of continuous variables was assessed using the Shapiro–Wilk test. For normally distributed variables, analysis of variance (ANOVA) was used to compare means between groups, while categorical variables were analyzed using Chi-square tests.

The risk of NAFLD was estimated using binary logistic regression analysis, with odds ratios (ORs) and 95% confidence intervals (CIs). Participants were categorized into four quartiles based on their LLDS scores, and these quartiles were included as categorical variables in the regression models. Trends across quartiles were assessed by treating the median LLDS score of each quartile as a continuous variable.

Before model fitting, assumptions of logistic regression were assessed. Linearity of continuous variables with the logit function was tested using the Box-Tidwell transformation, and multicollinearity was evaluated via variance inflation factors (VIF), ensuring all values were below 5. Adequacy of sample size was verified according to established recommendations for logistic regression, and residual diagnostics were examined for model fit validation. We adjusted the results using three regression models, progressively adjusting for energy, physical activity, and BMI (model 1), then additionally for waist circumference, education, vitamin D supplementation and age (model 2). Model comparison was conducted using Akaike Information Criterion (AIC) and Bayesian Information Criterion (BIC) to identify the best-fitting model. Additionally, predictive performance was evaluated using the AUC-ROC curve, with values exceeding 0.7 considered indicative of acceptable discrimination. The Hosmer-Lemeshow test was performed to confirm the goodness-of-fit. Logistic regression models were fitted using maximum likelihood estimation in SPSS software (version 20). Model parameters were estimated iteratively, and convergence criteria were met in all cases.

We adjusted for several key confounders, including age, BMI, physical activity, energy intake, waist circumference, education, and vitamin D supplementation. While participants with significant alcohol consumption were excluded to minimize its confounding effect, we acknowledge that residual confounding may still be present. Future studies with access to genetic data and comprehensive alcohol history are recommended to further validate these findings. We used linear regression to examine the relationship between the LLDS score and the novel anthropometric indices. In this study, *p*-values less than 0.05 were considered statistically significant, while p-values of 0.05, 0.06, and 0.07 were considered marginally significant.

## Results

### Characteristics of the study population

Table 1 summarizes the baseline characteristics of the study population, stratified by case and control groups, as well as the first and last quartiles of the LLDS. The mean age of participants in the NAFLD group was 49.34 ± 10.33 years, compared to 49.75 ± 9.85 years in the control group, with no significant age difference between the groups (*p* = 0.76). In terms of anthropometric variables, participants in the NAFLD group had significantly higher BMI (*p* = 0.023), waist circumference (*p* < 0.001), BRI (*p* < 0.001), and VAI (*p* = 0.01) than those in the control group. Biochemical tests revealed significant differences between the groups in liver enzyme levels, lipid profiles, and fasting blood sugar (FBS) levels (*p* < 0.05).

**Table 1:** Demographic, anthropometric, and lifestyle characteristics of NAFLD and non-NAFLD group as well as quartiles of LLDS.

Variables	Groups, mean (SD)		*p* value^a^	Quartiles of LLDS, mean (SD)		*p* value^a^
	Case (*n* = 180)	Control (*n* = 250)		Q1	Q4	
Age, y	49.34 (10.43)	49.75 (9.85)	0.76	49.53 (10.13)	49.42 (10.08)	0.87
BMI^b^, kg/m^2^	28.76 (5.62)	26.37 (4.39)	0.023	27.81 (4.69)	27.13 (5.18)	0.29
Waist- circumference (cm)	104.25 (13.54)	94.76 (10.27)	<0.001	101.15 (14.89)	95.15 (12.74)	0.004
ABSI	0.094 (0.03)	0.079 (0.02)	<0.001	0.088 (0.02)	0.085 (0.02)	0.21
BRI	8.23 (2.19)	6.04 (2.33)	<0.001	7.94 (2.85)	6.23 (1.53)	0.002
VAI	2.23 (0.56)	1.56 (0.39)	0.01	1.96 (0.53)	1.42 (0.45)	0.17
Physical activity (MET-min/week)	932.27 (426.19)	1043.37 (517.60)	0.38	954.18 (544.17)	987.38 (490.62)	0.62
Current smoker (yes), *n* (%)	32 (17.78)	41 (16.40)	0.59	21 (17.93)	18 (16.22)	0.71
SBP (mmHg)	124.65 (16.75)	123.39 (15.12)	0.47	123.86 (15.44)	123.65 (15.67)	0.75
DBP (mmHg)	78.83 (8.37)	76.19 (9.43)	0.25	77.41 (9.34)	76.95 (8.74)	0.37
ALT (mg/dl)	56.47 (26.19)	22.45 (15.29)	<0.001	41.25 (19.22)	34.57 (16.82)	0.03
AST (mg/dl)	35.27 (16.24)	21.19 (11.78)	<0.001	30.42 (13.27)	24.92 (9.48)	0.025
FBS (mg/dl)	108.17 (8.25)	93.41 (6.44)	<0.001	96.63 (7.46)	94.33 (7.15)	0.31
Triglyceride (mg/dl)	192.45 (16.34)	134.71 (13.56)	<0.001	176.35 (14.49)	153.23 (12.25)	0.034
Total cholesterol (mg/dl)	186.37 (37.52)	174.33 (34.25)	0.01	178.36 (36.24)	175.27 (34.22)	0.37
HDL (mg/dl)	40.60 (15.73)	48.24 (20.37)	<0.001	41.61 (17.63)	45.73 (19.35)	0.001
LDL (mg/dl)	124.35 (26.73)	107.42 (21.55)	<0.001	119.37 (25.75)	108.47 (20.73)	
Education status, *n* (%)						
Illiterate	13 (7.22)	20 (8)	0.48	7 (6.54)	6 (5.60)	0.75
Low education	98 (54.44)	140 (56)		58 (54.20)	60 (56)	
Higher education	69 (38.34)	90 (36)		42 (39.26)	41 (38.4)	

^aObtained from ANOVA for continuous variables and Chi-square for categorical variables. ABSI, A Body Shape Index; BRI, Body Roundness Index, VAI, Visceral Adiposity Index; ALT, alanine amino transferase; AST, aspartate amino transferase; FBS, fasting blood sugar; HDL, high-density lipoprotein; LDL, low-density lipoprotein; NAFLD, non-alcoholic fatty liver disease. MET, metabolic equivalents; SBP, systolic blood pressure; DBP, diastolic blood pressure; BMI, body mass index.^

### Dietary intake of study participants

Table 2 presents the dietary intake of participants across LLDS quartiles and between the case and control groups. The mean calorie intake in the NAFLD group was 2465.45 ± 701.23 kcal, significantly higher than the control group (2184.56 ± 679.15 kcal, *p* < 0.001). The NAFLD group also consumed significantly higher amounts of carbohydrates (*p* = 0.001) and fats (*p* < 0.001).

**Table 2:** Dietary intakes of study participants across case and control groups as well as quartiles of LLDS.

Variables	Groups, mean (SD)		*p* value^a^	Quartiles of LLDS, mean (SD)		*p* value^a^
	NAFLD (*n* = 180)	Control (*n* = 250)		Q1	Q4	
Energy (kcal/d)	2465.45 (701.23)	2184.56 (679.15)	0.004	2347.21 (683.24)	2289.55 (650.43)	0.25
Carbohydrate (g/d)	287.43 (75.19)	268.51 (69.43)	0.001	273.54 (71.36)	269.85 (67.43)	0.46
Protein (g/d)	78.45 (22.47)	76.92 (19.60)	0.59	73.24 (17.55)	79.41 (21.89)	<0.001
Fat (g/d)	85.42 (32.55)	74.67 (26.18)	<0.001	87.33 (34.32)	75.33 (25.46)	<0.001
SFA (g/d)	27.37 (9.43)	23.12 (8.51)	<0.001	27.88 (10.13)	24.70 (8.69)	<0.001
MUFA (g/d)	26.98 (12.47)	30.15 (14.30)	<0.001	31.63 (14.76)	25.31 (10.89)	<0.001
PUFA (g/d)	17.20 (8.10)	22.15 (9.43)	<0.001	23.44 (9.83)	17.37 (8.68)	<0.001
Cholesterol (mg/d)	270.14 (93.28)	253.59 (74.41)	0.001	272.30 (93.29)	263.21 (89.41)	<0.001
Fiber (g/d)	32.50 (16.79)	33.43 (14.76)	0.68	25.47 (14.33)	35.18 (17.36)	0.03
Calcium (mg/d)	1086.46 (259.55)	1113.40 (283.27)	0.36	923.15 (243.70)	1185.22 (261.70)	0.01
Iron (mg/d)	17.35 (6.34)	16.19 (5.83)	0.42	16.57 (6.22)	16.34 (5.93)	0.72
Zinc (mg/d)	10.53 (2.65)	11.38 (2.84)	0.23	10.32 (2.43)	12.40 (3.41)	0.025
Vitamin D (mcg/d)	2.01 (2.62)	2.78 (3.60)	0.042	1.65 (2.01)	2.89 (3.55)	<0.001
Vitamin E (mg/d)	15.34 (7.60)	19.30 (9.11)	0.001	17.46 (8.39)	18.15 (8.65)	0.28

^aObtained from ANOVA, SFA, saturated fatty acid; MUFA, Monounsaturated Fatty Acid; PUFA, polyunsaturated fatty acids.^

Additionally, participants with NAFLD consumed higher amounts of saturated fatty acids (SFA), total cholesterol, and lower amounts of monounsaturated fatty acids (MUFA) and polyunsaturated fatty acids (PUFA) compared to controls (*p* < 0.005). However, no significant differences were observed between the groups in vitamin D, vitamin E, or fiber intake. Across the quartiles of LLDS, participants in the higher quartiles consumed significantly more protein (*p* < 0.001), fiber (*p* = 0.03), calcium (*p* = 0.01), zinc (*p* = 0.025), and vitamin D (*p* < 0.001), while consuming less dietary fat, SFA, MUFA, PUFA, and total cholesterol (*p* < 0.05).

Table 3 displays the dietary intake of the 12 LLDS components (in grams per 1,000 kcal) across case and control groups and LLDS quartiles. Among the positive LLDS components, the NAFLD group consumed significantly lower amounts of vegetables, fruits, whole grains, legumes, and nuts (*p* < 0.05). For negative components, NAFLD participants consumed higher amounts of red and processed meat (*p* = 0.01) and sugar-sweetened beverages (*p* < 0.001). With increasing LLDS scores, the intake of positive components (except coffee and tea) rose significantly, while the intake of negative components declined significantly.

**Table 3:** Dietary consumption of the 12 components included in the Lifelines Diet Score (LLDS) in grams per 1,000 kcal among participants study (base case and control groups) and quartiles of LLDS.

Variables	Groups, mean (SD)		*p* value^a^	Quartiles of LLDS, mean (SD)		*p* value^a^
	NAFLD (*n* = 180)	Control (*n* = 250)		Q1	Q4	
LLDS score	32.49 (4.26)	39.34 (4.73)	<0.001	26.89 (3.61)	40.77 (3.92)	<0.001
Positive components						
Vegetables	108.45 (39.54)	124.57 (47.32)	<0.001	77.30 (23.66)	145.54 (41.29)	<0.001
Fruits	143.13 (52.73)	169.47 (71.37)	<0.001	135.90 (55.23)	197.42 (76.42)	<0.001
Whole grain products	28.47 (15.41)	36.77 (17.26)	0.001	21.45 (12.47)	42.24 (15.31)	<0.001
Legumes and nuts	10.43 (5.32)	15.32 (8.45)	<0.001	10.23 (4.33)	17.50 (6.73)	<0.001
Fish	4.24 (5.36)	4.95 (5.83)	0.25	3.19 (4.83)	6.79 (5.24)	<0.001
Oils and soft margarines	0.33 (0.15)	0.27 (0.18)	0.02	0.29 (0.17)	0.41 (0.22)	<0.001
Unsweetened dairy	179.24 (85.44)	183.48 (79.12)	0.35	132.90 (54.21)	212.36 (93.15)	<0.001
Coffee	0.03 (0.05)	0.033 (0.06)	0.74	0.02 (0.05)	0.03 (0.07)	0.29
Tea	1.37 (0.54)	1.33 (0.39)	0.69	1.25 (0.32)	1.41 (0.65)	0.38
Negative components						
Red and processed meat	11.83 (6.04)	9.35 (5.42)	0.01	13.70 (7.24)	8.82 (4.17)	<0.001
Butter, hard margarines	12.35 (7.30)	11.83 (6.47)	0.33	14.78 (9.19)	9.67 (5.23)	<0.001
Sugar-sweetened beverages	29.52 (15.37)	20.41 (13.49)	<0.001	35.62 (18.61)	17.19 (11.47)	<0.001

^aObtained from ANOVA.^

### Association of LLDS and odds of NAFLD

Table 4 presents the association between LLDS and NAFLD odds for the entire population and stratified by gender. In the crude model, participants in the highest LLDS quartile had 59% lower odds of NAFLD compared to the lowest quartile (OR = 0.41, 95% CI: 0.25–0.58; *p* < 0.001). In the fully adjusted model, higher LLDS scores were associated with 51% lower odds of NAFLD (OR = 0.49, 95% CI: 0.30–0.65; *p* < 0.001).

**Table 4:** Odds ratio (OR) and 95% confidence interval (CI) for NAFLD across the different quartiles of LLDS.

	LLDS Quartiles^∗∗^				P for trend
	Q1	Q2	Q3	Q4	
Total participants					
Crude model	1(Ref)	0.67 (0.44, 0.89)	0.55 (0.31, 0.71)	0.41 (0.25, 0.58)	<0.001
Model 1^∗^	1(Ref)	0.69 (0.45, 0.93)	0.59 (0.33, 0.78)	0.46 (0.27, 0.61)	<0.001
Model 2^†^	1(Ref)	0.75 (0.49, 1.15)	0.62 (0.34, 0.83)	0.49 (0.30, 0.65)	<0.001
Men					
Crude model	1(Ref)	0.70 (0.46, 0.98)	0.64 (0.42, 0.86)	0.58 (0.36, 0.72)	<0.001
Model 1^∗^	1(Ref)	0.73 (0.51, 1.10)	0.65 (0.43, 0.88)	0.62 (0.39, 0.75)	<0.001
Model 2^†^	1(Ref)	0.78 (0.55, 1.26)	0.67 (0.45, 0.92)	0.63 (0.40, 0.79)	<0.001
Women					
Crude model	1(Ref)	0.59 (0.34, 0.71)	0.47 (0.25, 0.64)	0.37 (0.24, 0.59)	<0.001
Model 1^∗^	1(Ref)	0.61 (0.36, 0.73)	0.53 (0.28, 0.68)	0.40 (0.27, 0.62)	<0.001
Model 2^†^	1(Ref)	0.65 (0.40, 0.81)	0.55 (0.33, 0.71)	0.45 (0.29, 0.64)	<0.001

^∗∗Binary logistic regression was used to obtain OR and 95% CI. ∗Model 1: adjusted for energy, physical activity, and BMI; †Model 2: Model 1 plus waist circumference, education, vitamin D supplementation and age.^

In subgroup analyses, male participants with higher LLDS scores had 42% lower odds of NAFLD in the crude model (OR = 0.58, 95% CI: 0.36–0.72; *p* < 0.001) and 37% lower odds in the adjusted model (OR = 0.63, 95% CI: 0.40–0.79; *p* < 0.001). Among female participants, those in the highest LLDS quartile had 63% lower odds of NAFLD in the crude model (OR = 0.37, 95% CI: 0.24–0.59; *p* < 0.001) and 55% lower odds in the adjusted model (OR = 0.45, 95% CI: 0.29–0.64; *p* < 0.001).

### Association of LLDS and some novel anthropometric indices

Table 5 summarizes the association between LLDS and novel anthropometric indices, including ABSI, BRI, and VAI. A significant inverse association was observed between LLDS scores and VAI (*β* = −1.37, 95% CI: −3.42 to −0.56; p-trend = 0.01). This relationship remained significant in the adjusted model (*β* = −1.14, 95% CI: −2.89 to −0.30; p-trend = 0.036). However, no significant associations were found between LLDS and either BRI or ABSI (*p* > 0.05).

**Table 5:** The association between LLDS and some novel anthropometric indices.

Anthropometric indices	Quartile of LLDS								P trend*
	Q1		Q2		Q3		Q4		
	*β* (95% CI)	*p* value*	*β* (95% CI)	*p* value*	*β* (95% CI)	*p* value*	*β* (95% CI)	*p* value*	
ABSI									
Crude	Ref	–	−0.086 (−0.38, 0.29)	0.58	−0.052 (−0.42, 0.33)	0.63	−0.12 (−0.25, 0.47)	0.34	0.64
Model 1	Ref	–	−0.097 (−0.35, 0.32)	0.74	−0.06 (−0.40, 0.33)	0.59	−0.09 (−0.18, 0.43)	0.66	0.59
Model 2	Ref	–	0.03 (−0.28, 0.34)	0.47	−0.083 (−0.36, 0.39)	0.71	0.001 (−0.001, 0.001)	0.89	0.77
BRI									
Crude	Ref	–	−0.35 (−0.76, 0.33)	0.38	−0.82 (−1.54, 0.25)	0.27	−1.37 (−3.42, −0.56)	0.013	0.01
Model 1	Ref	–	−0.39 (−0.83, 0.36)	0.41	−0.73 (−1.24, 0.33)	0.39	−1.30 (−3.15, −0.44)	0.014	0.025
Model 2	Ref	–	0.12 (−0.55,0.73)	0.44	−0.71 (−1.19, 0.41)	0.43	−1.14 (−2.89, −0.30)	0.021	0.036
VAI									
Crude	Ref	–	0.03 (−0.21, 0.12)	0.39	−0.05 (−0.38, 0.22)	0.31	−0.07 (−0.41, 0.11)	0.22	0.29
Model 1	Ref	–	0.06 (−0.19, 0.15)	0.204	−0.02 (−0.33, 0.27)	0.48	−0.06 (−0.39, 0.13)	0.47	0.37
Model 2	Ref	–	0.07 (−0.18, 0.17)	0.146	0.04 (−0.21, 0.15)	0.29	−0.03 (0.37, 0.18)	0.51	0.25

*Based on the linear regression test, Model 1: Adjusted for age, energy intake, and physical activity; Model 2: Model 1 further adjusted with education and vitamin D supplementation.

## Discussion

In this case–control study, we observed a significant inverse association between LLDS and the odds of NAFLD in adults. Higher LLDS scores, indicative of healthier dietary patterns, were linked to reduced NAFLD risk, even after adjusting for potential confounding factors such as age, BMI, and physical activity. These findings are consistent with prior research highlighting the protective role of high dietary quality in liver health (7). The LLDS emphasizes the consumption of fruits, vegetables, whole grains, and unsweetened dairy, which are recognized for their anti-inflammatory and antioxidant properties. These components likely improve metabolic profiles and reduce NAFLD risk.

Few studies have explored the relationship between LLDS and chronic disease risk. For example, Khadem et al. (12) found an inverse correlation between LLDS and metabolically healthy/unhealthy overweight and obesity (MHO/MUHO) phenotypes in a cross-sectional study, emphasizing LLDS’s positive effects against obesity, a key NAFLD risk factor. Nazari et al. examined the association between LLDS and anthropometric indices in Iranian women, finding no significant relationships with VAI, ABSI, or BRI (20).

The observed association between higher LLDS scores and reduced odds of NAFLD may be explained through several biological mechanisms linking diet quality to liver health. These pathways involve inflammation modulation, oxidative stress reduction, and improved lipid metabolism, all critical in NAFLD pathogenesis (11). High LLDS diets emphasize foods with potent anti-inflammatory properties, such as fruits, vegetables, whole grains, legumes, and nuts (10). These foods are rich in bioactive compounds like polyphenols and flavonoids, which inhibit pro-inflammatory cytokines (e.g., TNF-*α*, IL-6), thereby reducing liver inflammation and potentially preventing liver damage and fibrosis (21).

Oxidative stress is another major contributor to NAFLD progression, as it promotes hepatocyte injury and lipid peroxidation. The antioxidants in LLDS components, such as vitamins C and E and phytochemicals in fruits and vegetables, neutralize free radicals and support endogenous antioxidant defenses (22). This dual action helps prevent cellular damage and reduces fat accumulation in the liver (23).

Another mechanism through which LLDS may influence NAFLD is its impact on lipid metabolism. Dysregulated lipid metabolism is central to NAFLD, as excess triglycerides and free fatty acids accumulate in the liver, resulting in fat buildup and hepatic steatosis (24). The LLDS promotes the consumption of foods low in saturated fats and high in unsaturated fats, particularly monounsaturated and polyunsaturated fats found in fish, nuts, and certain oils. These unsaturated fats enhance lipid profiles by reducing triglyceride levels and increasing HDL cholesterol, thereby mitigating hepatic fat accumulation (25). Additionally, fiber-rich foods integral to the LLDS, such as whole grains and legumes, further support lipid metabolism by binding cholesterol and facilitating its excretion, reducing hepatic fat stores. LLDS components, including whole grains, legumes, and unsweetened dairy, are also linked to improved insulin sensitivity and glycemic control. Since insulin resistance plays a key role in NAFLD pathogenesis by increasing lipolysis in adipose tissue and delivering more free fatty acids to the liver, enhancing insulin sensitivity may significantly reduce NAFLD risk (9, 26).

On the other hand, the gut-liver axis has emerged as a crucial factor in the pathogenesis of NAFLD, with gut microbiota composition and function playing a significant role in liver health. Diet quality profoundly influences gut microbiota, and LLDS components, particularly dietary fiber, foster the growth of beneficial gut bacteria that produce short-chain fatty acids (SCFAs) (27). SCFAs possess anti-inflammatory properties and enhance the integrity of the intestinal barrier, thereby preventing the translocation of endotoxins that can trigger hepatic inflammation upon reaching the liver. By promoting a healthier gut microbiota, adherence to LLDS may help protect liver health and lower the risk of NAFLD (28).

This study has several notable strengths. Firstly, it is among the first to investigate the relationship between LLDS and NAFLD. Unlike prior studies that focused on specific nutrients or food groups, this research employs a comprehensive dietary quality index that encompasses both health-promoting and health-compromising dietary components. The findings highlight the utility of the LLDS as a practical tool for assessing dietary quality and informing nutritional interventions.

A key strength lies in the comprehensive dietary assessment, which utilized a validated FFQ covering 147 food items, enabling a detailed evaluation of participants’ dietary intake patterns. Additionally, the study accounts for key confounders, including physical activity and energy intake, which enhances the robustness and reliability of the results.

However, the study has several limitations. First, the hospital-based design and the recruitment of participants from a single hospital in Zanjan, Iran, limit the generalizability of the findings to other populations. Second, although alcohol consumption was excluded to minimize confounding effects, residual bias may still exist due to unmeasured lifestyle factors or genetic influences. Third, dietary intake was assessed using an FFQ, which is subject to recall bias. Participants may have inaccurately reported their food consumption, either due to memory lapses or social desirability bias, which could affect the accuracy of dietary intake and the LLDS. Finally, being a case–control study, it is inherently observational and cannot establish causal relationships. Future longitudinal or interventional studies are needed to further validate these findings and explore the mechanisms underlying these associations.

## Conclusion

This study demonstrates a significant inverse association between the LLDS and NAFLD, particularly in relation to anthropometric indices. Higher LLDS scores, reflecting better dietary quality, were linked to reduced odds of NAFLD and lower levels of visceral adiposity, as measured by indices such as the VAI and BRI. These findings suggest that dietary quality plays a crucial role in modulating body composition, particularly visceral fat, which is a key factor in the pathogenesis of NAFLD. The results highlight the potential of LLDS as an effective tool for assessing dietary quality and guiding nutritional interventions aimed at reducing NAFLD risk, especially by targeting visceral fat accumulation.

## Data Availability

The original contributions presented in the study are included in the article/supplementary material, further inquiries can be directed to the corresponding author.

## Footnotes

^1^ http://www.n4software.com
